# Analysis of differences in serological indicators between Graves’ disease and transient thyrotoxicosis during pregnancy

**DOI:** 10.20945/2359-4292-2026-0106

**Published:** 2026-09-12

**Authors:** Yuxin Jie, Gengxue Jin, Bo Sun, HuanHuan Zhang, Hui Chen

**Affiliations:** 1 Department of Endocrinology, Lanzhou University Second Hospital, Lanzhou, China; 2 Department of Endocrinology, Gansu Provincial Maternity and Child Health Care Hospital, Lanzhou, China

**Keywords:** Graves’ disease, gestational transient thyrotoxicosis, human chorionic gonadotropin, thyrotropin receptor antibodies, pregnancy

## Abstract

**Objective:**

To investigate serological differences between gestational Graves’ disease (GGD) and gestational transient thyrotoxicosis (GTT), and to establish practical cutoff values for differential diagnosis.

**Subjects and methods:**

We enrolled 97 first-trimester patients with gestational thyrotoxicosis (63 newly diagnosed GGD, 34 GTT) and 57 age- and gestational-age-matched healthy pregnant controls at Lanzhou University Second Hospital (China) between January 2020 and December 2024. We measured serum levels of TSH, FT4, FT3, TT4, TT3, TRAb, TgAb, TPOAb, and hCG and used ROC curve analysis to determine optimal diagnostic cutoff values and multivariable logistic regression to identify independent predictors.

**Results:**

Compared to the GTT group, the GGD group showed significantly higher TT3 (p = 0.033), TRAb (*p* < 0.001), and TPOAb (*p* = 0.002) levels, along with significantly lower hCG levels (*p* < 0.001). Compared to the healthy controls, the GGD group exhibited markedly suppressed TSH and elevated FT4, FT3, TT3, and TT4 (all *p* < 0.001). ROC analysis established the following optimal cutoffs: hCG > 63,518.50 IU/L (sensitivity 85.29%, specificity 53.97%), TT3 > 24.05 nmol/L (sensitivity 82.35%, specificity 57.14%), and WBC > 9.37 × 10^9^/L (sensitivity 70.59%, specificity 61.9%). Multivariable logistic regression identified hCG, TT3, TPOAb, and WBC as independent predictors distinguishing GGD from GTT.

**Conclusion:**

Our findings showed that TT3, TRAb, TPOAb, hCG, and WBC differed significantly between GGD and GTT and that the hCG cutoff of 63,518.50 IU/L provides practical diagnostic guidance that complements TRAb measurements.

## INTRODUCTION

Gestational thyrotoxicosis refers to a series of hypermetabolic symptoms caused by excessive secretion of thyroid hormones during pregnancy. The primary etiologies are gestational Graves’ disease (GGD) and gestational transient thyrotoxicosis (GTT). GGD is an autoimmune condition characterized by persistent activation of the thyroid-stimulating hormone (TSH) receptor by thyrotropin receptor antibodies (TRAb), independent of physiological negative feedback regulation (^1^,^2^). In contrast, GTT is typically associated with elevated human chorionic gonadotropin (hCG) levels in early pregnancy. Due to the structural similarity between hCG and TSH, hCG can weakly activate the TSH receptor, leading to a transient and mild increase in thyroid hormone levels, which remains partially regulated by negative feedback mechanisms (^3^,^4^). TRAb-mediated stimulation represents an uncontrolled pathological autoimmune attack, whereas hCG-mediated activation is a self-limiting physiological phenomenon. These two conditions therefore differ fundamentally in etiology, intensity, duration, and clinical significance (^5^,^6^).

Given their distinct pathogenic and pathophysiological mechanisms, clinical management and treatment strategies also differ significantly: GGD often requires antithyroid drug therapy and close monitoring, whereas GTT is primarily managed with supportive care (^7^–^9^). Consequently, accurate differential diagnosis between these conditions during early pregnancy is crucial for developing individualized treatment plans and improving maternal and fetal outcomes. This study aimed to systematically compare thyroid-related serological markers between patients with GGD and GTT, thereby providing a theoretical basis for precise clinical diagnosis and informed treatment decision-making.

## SUBJECTS AND METHODS

### Patients

A total of 97 first-trimester patients (<14 weeks’ gestation) with gestational thyrotoxicosis who presented to Lanzhou University Second Hospital between January 2020 and December 2024 were enrolled. Patients were divided into a GGD group (n = 63) and a GTT group (n = 34). The diagnostic criteria for GGD included positive TRAb, elevated free thyroxine (FT4), decreased TSH, and no prior history of hyperthyroidism prior to pregnancy. GTT was diagnostic criteria for GTT were negative TRAb, elevated FT4, decreased TSH, a self-limiting clinical course during follow-up, and no history of hyperthyroidism. Additionally, 57 healthy pregnant women, matched for age and gestational week and with normal thyroid function, were enrolled as control to provide a physiological reference baseline for pregnancy-related serological indicators.

### Inclusion and exclusion criteria

Eligible participants were first-trimester women with singleton pregnancies, decreased TSH, and elevated FT4 levels. Exclusion criteria were severe cardiovascular or cerebrovascular disease, significant autoimmune disorders, multiple gestation, conception via assisted reproductive technology, incomplete clinical data, or history of anti-thyroid drug therapy.

### Data collection

Comprehensive clinical data were gathered for all participants, including: (1) Baseline characteristics: age, gravidity, gestational age at delivery; (2) Laboratory parameters: white blood cell count (WBC), red blood cell count, hemoglobin, platelet count, neutrophil count, absolute neutrophil count, alanine aminotransferase, aspartate aminotransferase, total bilirubin, direct bilirubin, indirect bilirubin, albumin, triglycerides, total cholesterol, low-density lipoprotein cholesterol, blood urea nitrogen, creatinine, and uric acid; (3) Thyroid function tests: TSH, FT4, free triiodothyronine (FT3), total thyroxine (TT4), total triiodothyronine (TT3), TRAb, thyroid peroxidase antibody (TPOAb), and thyroglobulin antibody (TgAb); (4) Pregnancy-related hormone—hCG.

### Serological testing

Fasting venous blood samples (5 mL) were collected from all participants in the morning. Samples were centrifuged at 4000 rpm to separate serum, which was stored at -30 °C until analysis. Serum levels of TSH, FT3, FT4, TT3, TT4, TRAb, TPOAb, TgAb, and hCG were measured using an electrochemiluminescence immunoanalyzer (Cobas E602, Roche Diagnostics, Switzerland). Complete blood counts were analyzedz with an automated hematology analyzer (XN-9000, Sysmex, Japan), and biochemical parameters were assessed using an automated biochemistry analyzer (Cobas C701, Roche Diagnostics, Switzerland). The reference ranges for thyroid function tests are shown in **Table 1**.

**Table 1.** The reference range of thyroid function

Thyroid function indices (units)	Reference range
TT3, nmol/L	1.14–3.02
TT4, nmol/L	75.45–148.18
TSH, mIU/L	0.31–5.30
FT4, pmol/L	9.8–22.0
FT3, pmol/L	3.1–6.8
TRAb, IU/L	0–1.5
TPOAb, IU/mL	0–34.0
TgAb, IU/mL	0–115.0

### Statistical analysis

All statistical analyses were conducted using SPSS version 27.0. The normality of continuous variables was assessed with the Shapiro-Wilk test. Normally distributed data are presented as mean ± standard deviation and were compared between groups using independent samples t-tests. Non-normally distributed data are presented as median (interquartile range) and were compared using Mann-Whitney U tests. Categorical variables are expressed as counts (percentages) and were compared using Chi-square tests. Effect sizes were calculated using the non-parametric r metric derived from the Mann-Whitney U test (*r* = *Z*/*√N*). Optimal diagnostic cutoff values for hCG, WBC, and TT3 were determined by receiver operating characteristic (ROC) curve analysis, from which the corresponding sensitivity and specificity were obtained. A two-tailed *p-*value < 0.05 was considered statistically significant. Model calibration was assessed using the Hosmer-Lemeshow goodness-of-fit test, and Nagelkerke R^2^ was calculated to estimate the proportion of variance explained by the model.

### Ethical aspects

This research was approved by the Ethics Committee of The Second Hospital of Lanzhou University (no. 2025A-1286); all participants signed an informed consent form.

## RESULTS

### Comparison of baseline characteristics between the two groups

A total of 97 patients with gestational thyrotoxicosis were enrolled, comprising 34 in the GTT group and 63 in the GGD group, along with a control group of 57 healthy pregnant women with normal thyroid function (**Table 2**). No significant differences were observed between the GTT and GGD groups in baseline characteristics, including maternal age, gravidity and parity, mode of delivery, rate of preterm birth, complete blood count, liver and renal function, and lipid profiles (all *p* > 0.05), indicating that the groups were comparable. However, the WBC count was significantly higher in the GTT group compared to the GGD group (*Z* = -2.18, *p* = 0.029).

**Table 2.** Demographic and laboratory characteristics of the study subjects

Parameter	GTT (n = 34)	GGD (n = 63)	Control (n = 57)	t/z	*p*
RBC, ×10^12^/L	4.71 ± 0.54	4.85 ± 0.47	4.92 ± 0.39	-1.31	0.195
PLT, ×10^9^/L	264.06 ± 78.95	262.11 ± 72.31	264.79 ± 77.35	0.12	0.903
NEU, %	78.83 ± 5.03	79.34 ± 4.61	78.60 ± 7.06	-0.51	0.615
ANC, ×10^9^/L	7.76 ± 1.60	7.89 ± 1.34	8.14 ± 1.46	-0.42	0.676
ALB, g/L	44.69 ± 3.05	44.86 ± 4.16	44.14 ± 3.47	-0.21	0.834
DBIL, µmol/L	4.32 ± 1.61	4.28 ± 1.95	4.83 ± 1.90	0.09	0.928
TG, mmol/L	3.69 ± 1.64	3.46 ± 1.60	2.81 ± 1.31	0.66	0.513
LDL, mmol/L	2.89 ± 1.01	2.89 ± 1.12	2.62 ± 0.90	-0.00	0.997
TC, mmol/L	5.66 ± 1.47	5.56 ± 1.21	5.11 ± 1.10	0.37	0.713
Cr, µmol/L	48.82 ± 9.10	48.08 ± 13.33	46.91 ± 10.12	0.29	0.773
UA, µmol/L	297.94 ± 79.85	304.68 ± 76.04	299.98 ± 63.32	-0.41	0.683
Age, years	30.00 (28.00, 33.00)	30.00 (28.00, 32.00)	30.00 (28.00, 33.00)	-0.42	0.673
Gravidity	2.00 (1.00, 2.00)	1.00 (1.00, 2.00)	1.00 (1.00, 2.00)	-0.92	0.358
WBC, ×10^9^/L	9.49 (9.22, 9.71)	9.16 (8.53, 9.68)	9.22 (8.75, 9.55)	-2.18	0.029*
HGB, g/L	134.50 (96.50, 145.75)	138.00 (98.00, 144.00)	138.00 (117.00, 147.00)	-0.48	0.634
ALT, U/L	9.40 (8.30, 19.40)	9.40 (8.05, 13.80)	9.80 (8.40, 20.80)	-0.51	0.607
AST, U/L	20.85 (18.38, 30.23)	19.60 (15.45, 27.65)	21.30 (18.10, 28.10)	-1.02	0.307
TBIL, µmol/L	9.30 (8.53, 16.20)	9.30 (7.80, 14.15)	9.50 (7.90, 11.60)	-0.60	0.548
IBIL, µmol/L	8.50 (7.12, 9.28)	8.15 (7.10, 9.30)	8.70 (6.10, 9.30)	-0.27	0.789
BUN, mmol/L	3.90 (3.00, 4.30)	3.80 (3.20, 4.55)	3.70 (3.10, 4.30)	-0.24	0.812
Gestational age at delivery	39.00 (38.00, 40.00)	39.00 (38.00, 40.00)	39.00 (38.00, 39.00)	-0.53	0.594
Preterm, n (%)	4 (11.76%)	5 (7.94%)	6 (10.52%)	0.38	0.535

Normally distributed continuous data are expressed as mean ± standard deviation; non-normally distributed continuous data are expressed as median and interquartile range (Q1, Q3); Categorical data are expressed as n (%) and compared using Chi-square tests.

* *p* < 0.05 is considered statistically significant. Comparisons (P) provided are between GTT and GGD. ALB: albumin; ALT: alanine aminotransferase; ANC: absolute neutrophil count; AST: aspartate aminotransferase; BUN: blood urea nitrogen; Cr: creatinine; DBIL: direct bilirubin; HGB: hemoglobin; IBIL: indirect bilirubin; LDL: low-density lipoprotein cholesterol; NEU: neutrophil count; PLT: platelet count; RBC: red blood cell count; TBIL: total bilirubin; TC: total cholesterol; TG: triglycerides; UA: uric acid.

### Comparison of serum hCG, thyroid function, and autoantibody profiles among the three groups

Compared to the GTT group, the GGD group showed significantly higher levels of TT3 (*Z* = -2.13, *p* = 0.033), TRAb (*Z* = -8.10, *p* < 0.001), and TPOAb (*Z* = -3.10, *p* = 0.002), whereas hCG was significantly lower (*Z* = -4.42, *p* < 0.001) (**Table 3**). No significant differences were observed between the two groups in TT4, TSH, FT4, FT3, or TgAb (all *p* > 0.05). Compared to healthy controls, the GGD group exhibited markedly suppressed TSH (*Z* = -8.35, *p* < 0.001) and significantly elevated FT4 (*Z* = -8.35, *p* < 0.001), FT3 (*Z* = -9.42, *p* < 0.001), TT3 (*Z* = -7.43, *p* < 0.001), and TT4 (*t* = 9.41, *p* < 0.001). TRAb, TPOAb, and TgAb were also significantly higher in the GGD group than in controls (all *p* < 0.001). Notably, hCG levels did not differ between GGD patients and controls (*p* = 0.787).

**Table 3.** Comparison of hCG levels, thyroid function, and autoantibody titers in the study subjects

Parameter	GTT (n = 34)	GGD (n = 63)	Control (n = 57)	t/z (GTT vs. GGD)	P(GTT vs. GGD)	P(GGD vs. control)
TT4, nmol/L	199.54 ± 60.56	204.43 ± 75.65	106.29 ± 22.99	-0.32	0.746	<0.001*
hCG, mIU/L	125127.00 (74710.65, 200000.00)	60771.00 (18482.50, 102788.00)	50246.00 (24674.00, 88075.00)	-4.42	<0.001*	0.787
TSH, mIU/L	0.01 (0.01, 0.02)	0.01 (0.01, 0.04)	2.31 (1.68, 2.98)	-1.49	0.137	<0.001*
FT4, pmol/L	34.05 (28.32, 51.60)	31.80 (25.50, 42.30)	16.30 (11.70, 19.50)	-0.79	0.429	<0.001*
FT3, pmol/L	7.96 (6.63, 8.74)	7.61 (6.48, 9.20)	4.60 (3.30, 5.51)	-0.26	0.794	<0.001*
TT3, nmol/L	3.42 (3.16, 3.71)	3.95 (3.21, 5.39)	2.11 (1.47, 2.73)	-2.13	0.033*	<0.001*
TRAb, IU/L	1.00 (0.80, 1.12)	4.43 (2.40, 6.93)	-	-8.10	<0.001*	<0.001*
TPOAb, IU/mL	9.66 (9.00, 23.04)	34.40 (9.65, 98.05)	-	-3.10	0.002*	<0.001*
TgAb, IU/mL	34.50 (18.40, 137.62)	23.70 (16.25, 139.50)	-	-0.73	0.468	<0.001*

Normally distributed data are expressed as mean ± standard deviation, and non-normally distributed data are expressed as median (Q1, Q3).

* *p* < 0.05 is considered statistically significant.

### Effect sizes and bootstrap confidence intervals

TRAb demonstrated a large effect size (*r* = -0.82), while hCG and TPOAb showed medium effect sizes (*r* = -0.45 and *r* = -0.31, respectively) (**Table 4**). TT3 and WBC showed small-to-medium effect sizes (*r* = -0.22 for both). Bootstrap analysis with 1,000 resamples provided 95% confidence intervals for medians.

**Table 4.** Effect sizes and bootstrap 95% confidence intervals for medians

Parameter	Group	Median	95% CI (lower, upper)	Effect size (r)	Interpretation
hCG	GTT	125,127.00	(85,823.25, 163,864.00)	-0.45	Medium
	GGD	60,771.00	(25,933.60, 83,839.16)		
TRAb	GTT	1.00	(0.81, 1.10)	-0.82	Large
	GGD	4.43	(3.29, 6.00)		
TPOAb	GTT	9.66	(9.00, 19.80)	-0.31	Medium
	GGD	34.40	(19.85, 56.10)		
TT3	GTT	3.42	(3.26, 3.65)	-0.22	Small-to-medium
	GGD	3.95	(3.47, 4.46)		
WBC	GTT	9.49	(9.40, 9.62)	-0.22	Small-to-medium
	GGD	9.16	(8.85, 9.40)		

Effect sizes were calculated using the r metric (*r* = *Z*/*√N*). TRAb demonstrated a large effect size (*r* = -0.82), indicating a substantial difference between the GGD and GTT groups. Both hCG and TPOAb showed medium effect sizes (*r* = -0.45 and *r* = -0.31, respectively), whereas TT3 and WBC showed small-to-medium effect sizes (*r* = -0.22 for both).

### Diagnostic performance of serological parameters in differentiating new-onset GGD from GTT

**Figure 1.**
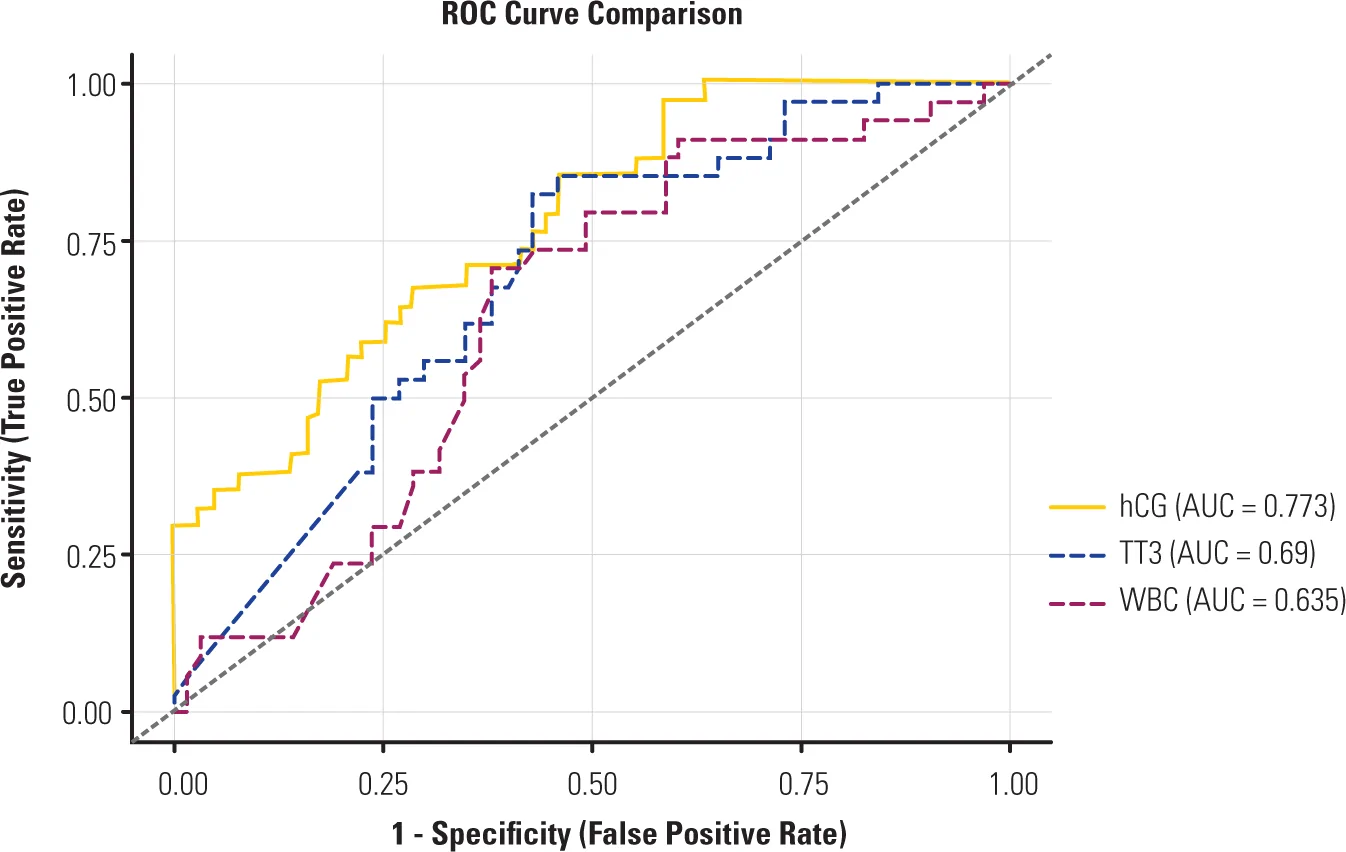
ROC curves of hCG, TT3, and WBC for differentiating between GGD and GTT patients.

The optimal cutoff values for TT3, WBC, and hCG in distinguishing GGD from GTT were determined using ROC curves, along with their corresponding areas under the curves (AUCs) (**Table 4**, **Figure 1**). For hCG, the optimal cutoff value was 63,518.50 (95% CI: 0.6794–0.8668), with a sensitivity of 85.29% and a specificity of 53.97%. For WBC, the optimal cutoff value was 9.37 (95% CI: 0.5218–0.7481), with a sensitivity of 70.59% and a specificity of 61.90%. For TT3, the optimal cutoff value was 24.05 (95% CI: 0.5847–0.7948), with a sensitivity of 82.35% and a specificity of 57.14%.

### Multivariable logistic regression analysis

To adjust for potential confounding factors, we conducted a multivariable logistic regression analysis with group assignment (GGD = 1, GTT = 0) as the dependent variable. The following variables were entered as covariates: hCG, TT3, TPOAb, and WBC. The Hosmer-Lemeshow test indicated good model fit (χ^2^ = 8.180, df = 8, *p* = 0.416), and the Nagelkerke R^2^ was 0.502, indicating that the model explained 50.2% of the variance in the outcome. Moreover, hCG, TT3, TPOAb, and WBC were all independent predictors of GGD vs. GTT (**Table 5**). Higher hCG levels were associated with lower odds of GGD (OR = 1.000 per IU/L increase, 95% CI: 1.000–1.000, *p* < 0.001), reflecting the markedly elevated hCG levels in GTT patients. Higher TT3 levels were associated with increased odds of GGD (OR = 1.569, 95% CI: 1.051–2.344, *p* = 0.028). TPOAb positivity was associated with increased odds of GGD (OR = 1.010, 95% CI: 1.001–1.019, *p* = 0.026). In contrast, higher WBC count was associated with lower odds of GGD (OR = 0.559, 95% CI: 0.321–0.973, *p* = 0.040), consistent with the observation that GTT patients had significantly higher WBC counts in univariate analysis. The results of the multivariable logistic regression analysis are presented in **Table 6**.

**Table 5.** ROC curves and cutoff values of hCG, WBC, and TT3 for differentiating GGD from GTT

Variables	Cutoff value	AUC	95% CI	Sensitivity (%)	Specificity (%)
hCG	63518.50	0.773	0.6794–0.8668	85.29	53.97
WBC	9.37	0.635	0.5218–0.7481	70.59	61.9
TT3	24.05	0.690	0.5847–0.7948	82.35	57.14

**Table 6.** Multivariable logistic regression analysis for predicting GGD (vs. GTT)

Variable	B	SE	Wald	*p*	OR	95% CI for OR	
						Lower	Upper
hCG	0.000	0.000	15.110	<0.001	1.000	1.000	1.000
TT3	0.451	0.205	4.852	0.028	1.569	1.051	2.344
TPOAb	0.010	0.004	4.931	0.026	1.010	1.001	1.019
WBC	-0.581	0.283	4.226	0.040	0.559	0.321	0.973
Constant	5.377	2.741	3.847	0.050	216.342	-	-

The variables entered were hCG, TT3, TPOAb, and WBC. TRAb was not included due to complete separation between groups (positive in all GGD and negative in all GTT). Model fit: Hosmer-Lemeshow χ^2^ = 8.180, df = 8, *p* = 0.416; Nagelkerke R^2^ = 0.502. CI: confidence interval; OR: odds ratio; SE: standard error.

## DISCUSSION

Accurate differentiation between GGD and GTT is central to the clinical management of gestational thyrotoxicosis. Among currently available serological markers, substantial evidence has established the critical role of TRAb in the differential diagnosis of GGD and GTT (^1^,^2^,^5^). However, the diagnostic utility of other serum parameters warrants further investigation (^10^,^11^). Our findings confirmed the critical role of TRAb in distinguishing GGD from GTT and further demonstrates the significant contributions of TT3, TPOAb, WBC, and hCG to this diagnostic distinction.

TRAb is a group of polyclonal antibodies that directly target the TSH receptor on the membrane of thyroid follicular cells and belong to the immunoglobulin G class. Based on their functional effects on the TSH receptor, TRAb is mainly classified into three subtypes: Stimulating antibodies, which mimic the biological activity of TSH and, upon binding to the TSH receptor, activate the cAMP signaling pathway, continuously stimulating thyroid follicular cell proliferation and the synthesis and secretion of thyroid hormones. This is the primary pathogenic antibody responsible for hyperthyroidism in Graves’ disease (^12^). Blocking antibodies bind to the TSH receptor but do not activate the cAMP pathway; rather, they block the binding of TSH or stimulating antibodies to the receptor, exerting an antagonistic effect that can result in hypothyroidism. Neutral antibodies can bind the receptor but do not affect its function and do not directly impact thyroid activity (^1^).

International guidelines recommend measuring TRAb when suppressed serum TSH is detected in the first trimester to clarify the etiology of thyrotoxicosis (^6^,^7^). Timely TRAb testing not only aids in confirming the diagnosis of newly onset Graves’ disease during pregnancy but also guides medication management, assesses treatment response, and evaluates the risk of fetal and neonatal hyperthyroidism, thereby enabling targeted monitoring of high-risk pregnancies (^13^–^15^). Danda and cols. (^16^) reported that serum TRAb levels were significantly higher in the GGD group than in the GTT group (*p* < 0.0001), further supporting the utility of TRAb as a discriminatory biomarker in the differential diagnosis of pregnancy-related thyrotoxicosis.

In our investigation, serum TRAb levels were significantly elevated in the GGD group relative to both the GTT group (*Z* = -8.10, *p* < 0.001) and the healthy control cohort (*p* < 0.001). Drawing on the most current clinical data, our results not only corroborate established guidelines and prior literature regarding the robust discriminatory capacity of TRAb in distinguishing GGD from GTT and healthy controls but also provide a solid evidence-based foundation for its clinical application in differential diagnosis. Moreover, the marked divergence of TRAb levels in GGD patients compared to normophysiological controls in this real-world dataset emphasizes the substantial association between TRAb dynamics and the pathophysiology of GGD.

In terms of thyroid autoantibodies, our study found that TPOAb levels were significantly higher in the GGD group than in the GTT group and the healthy control group. Although TPOAb is traditionally associated with Hashimoto’s thyroiditis, it is frequently positive in patients with Graves’ disease, reflecting a broader autoimmune diathesis involving the thyroid gland (^17^). In pregnancy, a state characterized by complex immunological adaptation, the presence of TPOAb may further support an underlying autoimmune etiology (^18^), thereby aiding differentiation from GTT, which is driven primarily by hCG-mediated thyroid stimulation rather than autoimmunity (^19^). Moreover, TPOAb positivity in pregnant women with Graves’ disease has been linked to an increased risk of postpartum thyroid dysfunction, warranting continued follow-up after delivery (^20^,^21^).

The more pronounced TT3 elevation observed in GGD patients compared to those with GTT reflects fundamental differences in thyroid hormone metabolism between these two conditions. In Graves’ disease, sustained activation of the TSH receptor by TRAb not only increases thyroid hormone synthesis but also upregulates type 1 iodothyronine deiodinase (DIO1) within the thyroid gland. DIO1 catalyzes the outer-ring deiodination of thyroxine (T4) to the biologically active triiodothyronine (T3), resulting in preferential T3 secretion and an elevated T3:T4 ratio (^22^). In contrast, GTT patients typically exhibit elevated serum T4 levels without a proportional increase in T3, as GTT is caused by stimulation of the thyroid TSH receptor by high levels of hCG in maternal circulation (^23^). The thyroid hormone secretion pattern in GTT is characterized by a predominant increase in T4, with only a mild or disproportionate elevation of T3 (^24^). The distinct mechanisms of TT3 elevation in GTT and GGD are summarized in **Table 7**.

**Table 7.** Distinct mechanisms of TT3 elevation in GTT vs. GGD

Parameter	GTT	GGD
Stimulus	hCG (placental hormone)	TRAb (autoantibody)
Duration	Transient (first trimester)	Persistent throughout pregnancy
TSH Receptor activation	Weak, transient	Strong, sustained
DIO1 Activity	Mildly increased or unchanged	Markedly upregulated
T4-to-T3 Conversion	Minimal enhancement	Enhanced (outer-ring deiodination)
T3 Secretion pattern	Proportional to T4 (normal T3:T4 ratio)	Preferential (high T3:T4 ratio)
Clinical correlation	Relatively lower TT3 levels	Higher TT3 levels

hCG is a hormone produced by the placenta during pregnancy. Both hCG and TSH belong to the glycoprotein hormone family, which also includes luteinizing hormone and follicle-stimulating hormone. These hormones share a common alpha subunit and possess structurally similar beta subunits that confer receptor specificity (^25^). Their receptors (i.e., TSH and luteinizing hormone/chorionic gonadotropin) also exhibit high homology, particularly in the extracellular domains, allowing hCG to possess weak thyroid-stimulating activity (^26^). Elevated hCG levels tend to be associated with hyperemesis gravidarum (^10^). Studies have shown a positive correlation between hCG and free T4 levels in patients tested at 8–11 weeks of gestation (^27^). Therefore, in early pregnancy, clinicians should first suspect GTT in cases of thyrotoxicosis with extremely high hCG, elevated T4, and hyperemesis gravidarum. Conversely, a more pronounced elevation in T3 should strongly suggest GDD (^11^).

This study identified a significantly higher WBC count in the GTT group compared to the GGD group at baseline. Physiologic leukocytosis during pregnancy is a recognized phenomenon, attributed to enhanced inflammatory responses. This adaptation may result from a complex interplay of selective immune tolerance, immunomodulation, and immunosuppressive mechanisms necessary to sustain a successful pregnancy (^28^,^29^). The observed elevation in the GTT group may reflect a more pronounced systemic inflammatory or stress state facilitated by markedly high hCG levels.

We evaluated the diagnostic value of hCG, WBC, and TT3 in differentiating GGD from GTT using ROC curve analysis. The results showed that hCG demonstrated superior diagnostic performance (AUC = 0.852), with an optimal cutoff value of 63,518.5 IU/L and a sensitivity of 85.29%, indicating strong discriminatory ability in screening for GTT. In contrast, WBC and TT3 exhibited moderate diagnostic utility (AUC = 0.712 and 0.698, respectively), with relatively lower specificities (61.9% and 57.14%). This can be explained by the limited diagnostic specificity of both markers: although WBC may be elevated in Graves’ disease, it is a nonspecific marker of inflammation, and total triiodothyronine can be elevated in both conditions due to the hyperthyroid state itself, limiting their ability to effectively distinguish between them (^24^,^30^). In clinical practice, when evaluating pregnant patients with thyrotoxicosis, an hCG level markedly above this cutoff, in the context of negative or low-titer TRAb, strongly suggests GTT. This approach helps to avoid premature initiation of antithyroid drug therapy and reduces potential risks to the fetus (^31^). However, due to the diagnostic limitations of WBC and TT3, reliance on either parameter alone is not recommended. Instead, a comprehensive assessment based on a combination of laboratory parameters and clinical findings, especially TRAb levels, as well as the symptoms presented by the patient.

This study has several limitations that should be acknowledged. First, as a single-center retrospective study, it may be subject to selection bias, and the generalizability of the findings is limited. The proposed diagnostic cutoff values should be externally validated before being applied to other populations or to different medical settings. Second, the relatively small sample size, especially the small number of GTT cases, may have influenced statistical power and the detection of subtle group differences. Third, although all subjects were in the first trimester, gestational age at blood collection was not completely uniform, raising the possibility of residual confounding due to differences in pregnancy stage. Lastly, the lack of follow-up data on postpartum thyroid function and long-term maternal and neonatal outcomes precludes assessment of the predictive value of maternal serologic markers for long-term outcomes. Future research should involve multicenter, prospective, large-scale cohort studies with systematic postpartum follow-up to further validate these results and their clinical applicability.

In conclusion, this study further confirms that TRAb, as a key test recommended by current guidelines, holds core diagnostic value in identifying the etiology of gestational thyrotoxicosis. The observed differences in TPOAb, hCG, and TT3 levels provide valuable ancillary evidence to help determine the underlying cause. For patients with thyrotoxicosis of unknown origin, TRAb testing should be prioritized. A positive TRAb result, especially in conjunction with positive TPOAb, strongly suggests GGD and warrants the initiation of antithyroid drug therapy and close monitoring. In contrast, a negative TRAb result combined with significantly elevated hCG levels supports a diagnosis of GTT, for which symptomatic management and vigilant observation represent a reasonable approach. This serology-based diagnostic algorithm facilitates timely intervention in GGD while helping to avoid unnecessary antithyroid treatment in GTT, thereby offering a strategy to optimize pregnancy management. Its ultimate impact on maternal and neonatal outcomes requires further validation in prospective studies.

## Data Availability

the datasets used and/or analyzed during the current study are available from the corresponding author upon reasonable request.
